# Construction and systematic evaluation of a machine learning-based cuproptosis-related lncRNA score signature to predict the response to immunotherapy in hepatocellular carcinoma

**DOI:** 10.3389/fimmu.2023.1097075

**Published:** 2023-01-25

**Authors:** Dingyu Lu, Jian Liao, Hao Cheng, Qian Ma, Fei Wu, Fei Xie, Yingying He

**Affiliations:** ^1^ Oncology Department, Deyang People’s Hospital, Deyang, China; ^2^ Intensive care Unit, Deyang People’s Hospital, Deyang, China

**Keywords:** cuproptosis-related lncRNA score, hepatocellular carcinoma, machine learning, prognostic model, immunotherapy

## Abstract

**Introduction:**

Hepatocellular carcinoma (HCC) is a common malignant cancer with a poor prognosis. Cuproptosis and associated lncRNAs are connected with cancer progression. However, the information on the prognostic value of cuproptosis-related lncRNAs is still limited in HCC.

**Methods:**

We isolated the transcriptome and clinical information of HCC from TCGA and ICGC databases. Ten cuproptosis-related genes were obtained and related lncRNAs were correlated by Pearson’s correlation. By performing lasso regression, we created a cuproptosis-related lncRNA prognostic model based on the cuproptosis-related lncRNA score (CLS). Comprehensive analyses were performed, including the fields of function, immunity, mutation and clinical application, by various R packages.

**Results:**

Ten cuproptosis-related genes were selected, and 13 correlated prognostic lncRNAs were collected for model construction. CLS was positively or negatively correlated with cancer-related pathways. In addition, cell cycle and immune related pathways were enriched. By performing tumor microenvironment (TME) analysis, we determined that T-cells were activated. High CLS had more tumor characteristics and may lead to higher invasiveness and treatment resistance. Three genes (*TP53*, *CSMD1* and *RB1*) were found in high CLS samples with more mutational frequency. More amplification and deletion were detected in high CLS samples. In clinical application, a CLS-based nomogram was constructed. 5-Fluorouracil, gemcitabine and doxorubicin had better sensitivity in patients with high CLS. However, patients with low CLS had better immunotherapeutic sensitivity.

**Conclusion:**

We created a prognostic CLS signature by machine learning, and we comprehensively analyzed the signature in the fields of function, immunity, mutation and clinical application.

## Introduction

Hepatocellular carcinoma (HCC) ranks fifth in most common carcinoma and second in cancer-related death (1). As a major histological type, HCC is identified by a high mortality rate and rapid progression (2). The main treatments for early and advanced HCC include surgical resection, multi-kinase inhibitors and immunotherapy. However, the therapeutic effect was limited due to the treatment resistance or adverse reactions (3–5). Therefore, it is vital to individually predict the overall survival rate and sensitivity of the drugs to guide clinical treatment and improve the therapeutic effect for HCC patients.

Cuproptosis is an innovative cell death pathway in which copper can directly bind to the tricarboxylic acid (TCA) cycle and cause protein stress, which eventually results in cell death (6). Copper, which is essential for life, plays a vital role in regulating homeostasis. Lack of copper may cause dysfunction of copper-binding enzymes. However, increasing the level of copper may lead to cell death (7). A recent study revealed that the level of intracellular copper may regulate the progression of cancer (8). Thus, increasing the accumulation of intracellular cancer is considered to be a novel therapeutic target for cancer cell killing (9). According to the mechanism, it is necessary to determine the regulators of the novel form of cell death in HCC patients.

Long noncoding RNAs (lncRNAs) consist of more than 200 nucleotides and mostly do not encode proteins (10). The functions of some lncRNAs have been widely studied, and they are involved in regulating chromatin dynamics, genes, cell differentiation, growth and development (11). Thanks to next-generation sequencing, thousands of lncRNAs have been revealed to be abnormally expressed in various cancers (12). Most importantly, many lncRNAs were associated with prognosis in many types of cancer as well as potential therapeutic targets (13–15).

In our study, we constructed a novel machine learning-based cuproptosis-related lncRNA prognostic signature for HCC patients with bioinformatic analysis. We performed functional, immune and mutational analyses to comprehensively evaluate the created model. Moreover, our model can guide the clinical treatment with satisfactory results.

## Methods

### Data extraction

Ten cuproptosis-related genes were obtained from a previous article. The related data, including transcriptome RNA sequencing and clinical data, were extracted from The Cancer Genome Atlas (TCGA) (https://portal.gdc.cancer.gov/) and International Cancer Genome Consortium (ICGC) (https://dcc.icgc.org) online databases. Patients in both datasets were collected based on the following criteria: (a) pathological diagnosed with LIHC (Liver hepatocellular carcinoma); (b) available clinical information (including age, gender, stage, and complete follow-up information); (c) available gene expression matrix. Finally, we collected 340 patients in the TCGA-LIHC cohort and 226 patients in the ICGC-LIHC cohort. The cohort of DNA methylation and copy number were obtained from UCSC Xena (https://xena.ucsc.edu/), which belongs to University of California Santa Cruz.

### Establishment of the cuproptosis-related prognostic lncRNA signature

We explored the correlation between 10 cuproptosis-related genes and lncRNAs by performing Pearson’s correlation with a P-value < 0.05. The network was constructed by R the package “Igraph”. To filter the prognostic lncRNAs and establish the cuproptosis-related prognostic lncRNA signature, we performed LASSO regression. The corresponding coefficients (β) of the signature were obtained. The cuproptosis-related lncRNA score (CLS) was calculated by the following formula: CLS = ∑ [expression (cuproptosis-related prognostic lncRNA signature)*β]. The cutoff value was the median CLS value in each data set.

### Validation of the cuproptosis-related prognostic lncRNA signature

We constructed the lncRNA signature by using the TCGA dataset as the training cohort. Afterward, the ICGC dataset was used for validation as the testing cohort. To evaluate the capacity of prediction, we calculated the concordance index (C-index) by using the R package “Pec”. The area under the curve (AUC) analysis was obtained to assess the reliability of our signature with the R package “timeROC”. The heatmap was created by the R package “pheatmap”. Kaplan-Meier (K-M) analysis was performed in TCGA and ICGC cohorts with the R package “survival”.

### RNA isolation and RT-qPCR

We isolated RNA using an RNeasy Mini Kit (QIAGEN, Hilden, Germany). The RNA was reversed to cRNA by utilizing a High-Capacity RNA-to-cDNA^TM^ Kit (Thermo Fisher Scientific, Hilden, Germany). Afterward, we performed RT-qPCR with PowerUp^TM^ SYBR^TM^ Green Master Mix (Thermo Fisher Scientific, Hilden, Germany) based on the manufacturer’s instructions. The sequences of the lncRNA primers are shown (**Table S2**). The relative expression was calculated using the 2^-ΔΔCt^ method.

### Nomogram establishment based on CLS

We performed the univariate Cox regression and multivariate Cox regression with the R package “survival”. To individually predict the overall survival rate, we established a CLS-based nomogram according to the Cox regression analysis by the R package “RMS”. Then, we obtained the calibration curves and AUCs by utilizing the R packages “rms” and “survivalROC” respectively. Moreover, the decision curve analysis (DCA) was analyzed with the R package “rmda” to further evaluate the superiority of the nomogram.

### Functional and immune analyses

The correlation heatmap was analyzed by the R package “ggcor”. After obtaining the differentially expressed genes, we introduced an online resource called Metascape (https://metascape.org) to determine the enrichment items. Gene set enrichment analysis (GSEA) was used to analyze the enriched pathways. The immune-correlated pathways were isolated from a previous article (16). Other pathways of interest were obtained from a published article (17). We obtained the homologous recombination deficiency (HRD) score, cancer-testis antigen (CTA) score and intratumor heterogeneity from an article (18). The R package “cibersortR” was utilized to obtain the relative abundance of each tumor-infiltrating immune cell (TIC) in each sample. Moreover, the tumor microenvironment was analyzed by ESTIMATE algorithm.

### Mutational analyses

The mutational data were extracted from the TCGA using the R package “TCGAbiolinks”. We created the mutational waterfall plot and the lollipop chart with the R package “maftools”. The tumor mutational burden (TMB) of each sample was calculated. Furthermore, the mutational spectrum of mutational signatures was determined based on the R package “MutationalPattern”.

### Clinical decision based on CLS

The genomics of drug sensitivity in cancer (GDSC) database (www.cancerRxgene.org) was introduced. The half-maximal inhibitory concentration (IC50) was calculated with the R package “pRRophetic”. The immunophenoscore (IPS) was calculated with a reported algorithm (19). We performed subclass mapping analysis (20) to assess the response to PD-1 and CTLA4 in an existing dataset containing comprehensive immunotherapy information in melanoma patients (21).

The response to immunotherapy was detected by tumor immune dysfunction and exclusion (TIDE) mode (http://tide.dfci.harvard.edu) (22). Five biomarkers, including IPS, interferon gamma (IFNG), CD274, CD8 and myeloid-derived suppressor cell (MDSC), were compared with CLS to evaluate the accuracy of prediction according to the AUC analyses. In addition, the database ConnectivityMap (https://clue.io/) was utilized to figure out the potential small molecule drugs and the corresponding mechanism of action.

### Statistical analyses

R software (version 4.0.4) was used for all statistical analyses. Adobe Illustrator was used for managing all figures. We performed the correlation analyses by Pearson’s correlation. The Wilcoxon test was used to analyze the difference between two groups. The proportion of the data was evaluated *via* the chi-squared test. A P-value less than 0.05 was considered to be significant. *P < 0.05, **P < 0.01, ***P < 0.001, ****P < 0.0001.

## Results

### Ten cuproptosis-associated genes and related lncRNAs were identified

**Figure 1:**
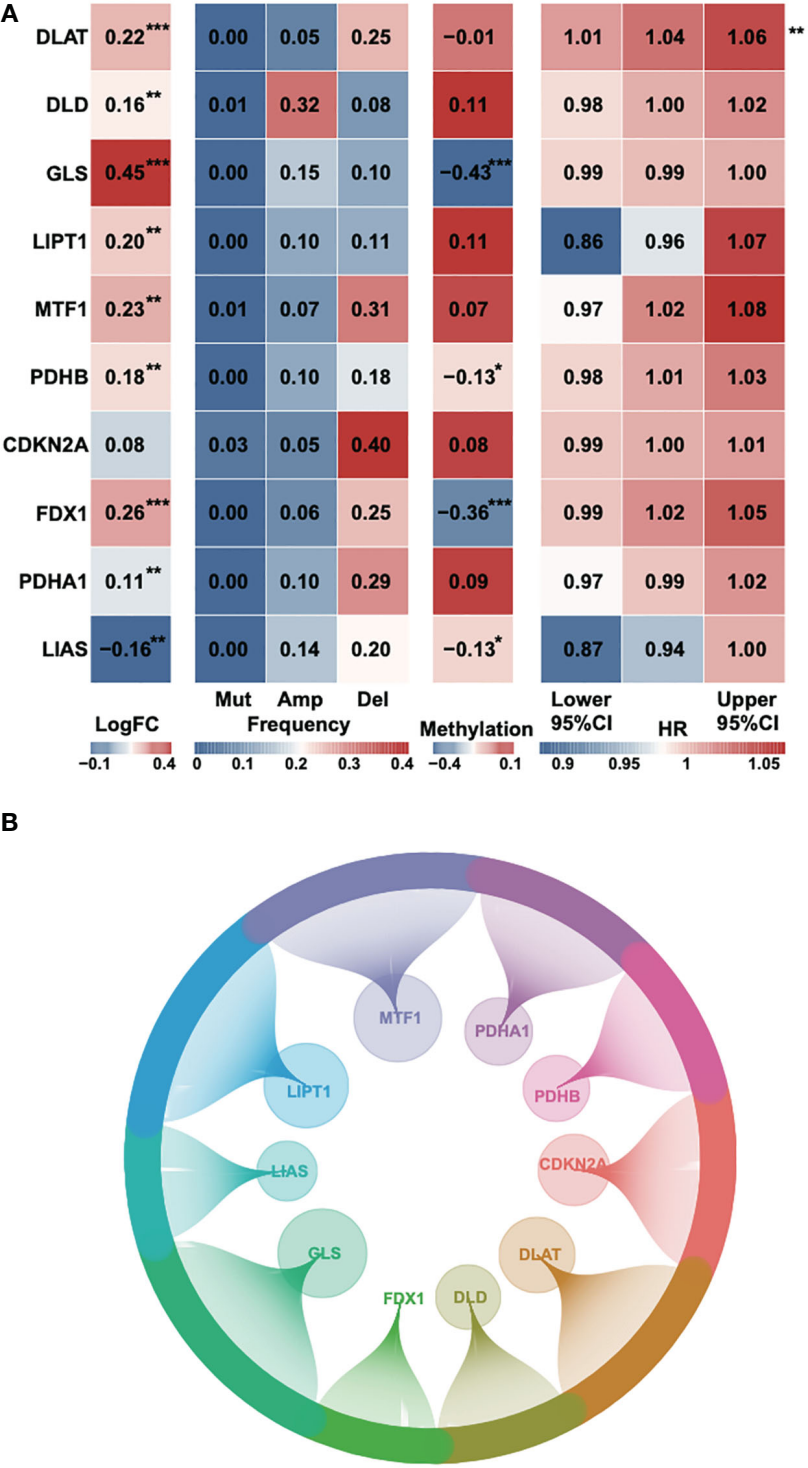
Identification of cuproptosis-related genes and corresponding lncRNAs. **(A)** The fold change, mutational frequency, methylation level and Hazard ratio of the ten cuproptosis-related genes. **(B)** The correlated lncRNAs of the ten cuproptosis-related genes. *P-value < 0.05, **P-value < 0.01, ***P-value < 0.001.

According to a recent high-quality article (6), we collected 10 cuproptosis-associated genes for further research (**Table S1**). First, we analyzed the fold change, mutational frequency, methylation and hazard ratio of ten cuproptosis-associated genes (**Figure 1A**). *DLAT*, *DLD*, *GLS*, *LIPT1*, *MTF1*, *PDHB* and *FDX1* were highly expressed in HCC, while *PDHA1* and *LIAS* were downregulated in HCC. *CDKN2A* was considered to be the most frequently mutated gene. The lowest methylation level was found in the *GLS* gene. *DLAT* was found to be a risk factor in HCC. Afterward, we performed Pearson’s correlation to identify 242 correlated lncRNAs with a P-value < 0.05, and the result was exhibited using a circle plot (**Figure 1B**). Two hundred and twenty four lncRNAs were selected.

### Construction of a prognostic signature based on 13 cuproptosis-related lncRNAs

**Figure 2:**
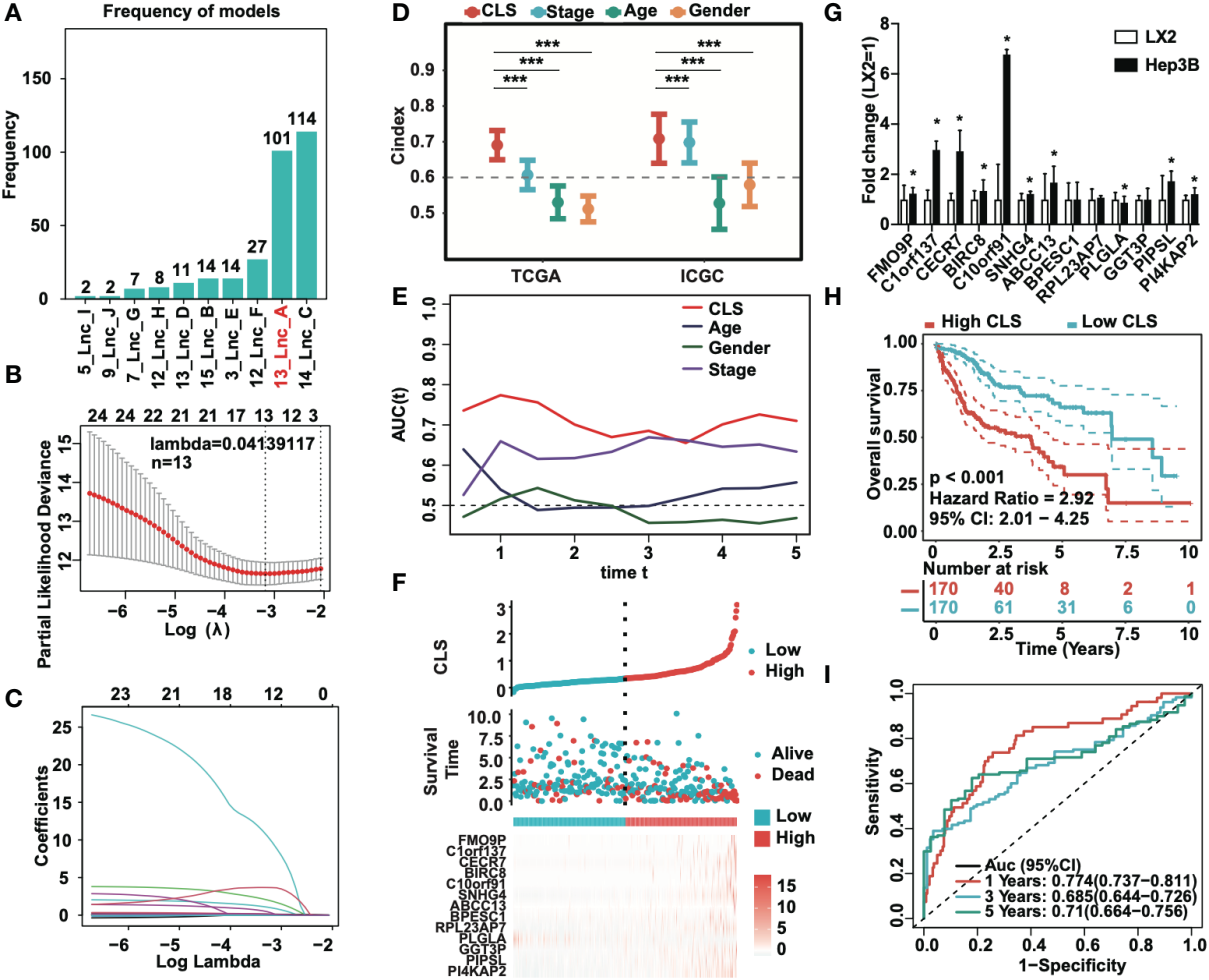
Prognostic signature based on CLS was created. **(A)** Lasso regression of the cuproptosis-related lncRNAs. **(B)** Identification of the tuning parameter in Lasso model. **(C)** The coefficients in Lasso model. **(D)** The C-index of CLS, stage, age and gender in TCGA and ICGC databases. **(E)** The AUC of CLS, age, gender and stage in TCGA. **(F)** The survival status and the expression of the 13 cuproptosis-related lncRNAs of each sample ranked from high to low CLS. **(G)** The mRNA expression of 13 cuproptosis-related lncRNAs in HCC cell line Hep3B compared to the hepatic stellate cell line LX2. **(H)** Kaplan-Meier analysis of the high and low CLS patients. **(I)** The 1-, 3- and 5-year AUC of the prognostic signature. *P-value < 0.05, ***P-value < 0.001.

To identify the most stable prognostic model, we performed Lasso regression and revealed that the 13-lncRNA and 14-lncRNA models were suitable for prognostic signature construction. Since only one lncRNA was not included in the 13-lncRNA model, we eventually selected the 13-lncRNA model as the principle of simplicity (**Figure 2A**). The lasso regression model of the 13 lncRNAs (lambda=0.04139117) is shown (**Figure 2B**). Then, we performed ridge regression and obtained the same result (**Figure 2C**). In addition, we introduced a new scoring system, the cuproptosis-related lncRNA score (CLS), to evaluate the risk level in HCC. By detecting the C-index, which is used for the assessment of prediction capacity and reliability (23), we uncovered that the C-index was the highest in CLS compared to stage, age and sex in both TCGA and ICGC databases (**Figure 2D**). The results illustrated that CLS may act as a suitable signature with a high prediction capacity in HCC. Furthermore, we also performed AUC analysis to evaluate our model in TCGA and ICGC datasets (**Figures 2E**, **S1A**), and the results indicated that CLS was better than some traditional prediction markers. Then, we calculated the CLS in each sample and ranked the order from low to high CLS. The survival status and the expression of 13 lncRNAs in each sample are illustrated in both datasets (**Figures 2F**, **S1B**). The results revealed that high CLS patients obtained a worse survival status, and that most lncRNAs in our model were highly expressed in high CLS patients except PLGLA. Afterward, we performed the RT-qPCR to detect the mRNA expression of 13 lncRNAs in the LX2 hepatic stellate cell line and Hep3B HCC cell line (**Figure 2G**). In addition, we pointed out that the overall survival (OS) rate was lower in high CLS patients by performing Kaplan-Meier analysis in the TCGA and ICGC databases (P < 0.001) (**Figures 2H**, **S1C**). We subsequently performed AUC analysis to assess the accuracy of our CLS system, the AUCs at 1-, 3-, and 5-year were 0.774, 0.685 and 0.71, respectively, in the TCGA database (**Figure 2I**) and 0.692, 0.729 and 0.903, respectively, in the ICGC database (**Figure S1D**), which showed that our CLS system was satisfactory for prognostic prediction.

### Establishment of a CLS-based nomogram for HCC

**Figure 3:**
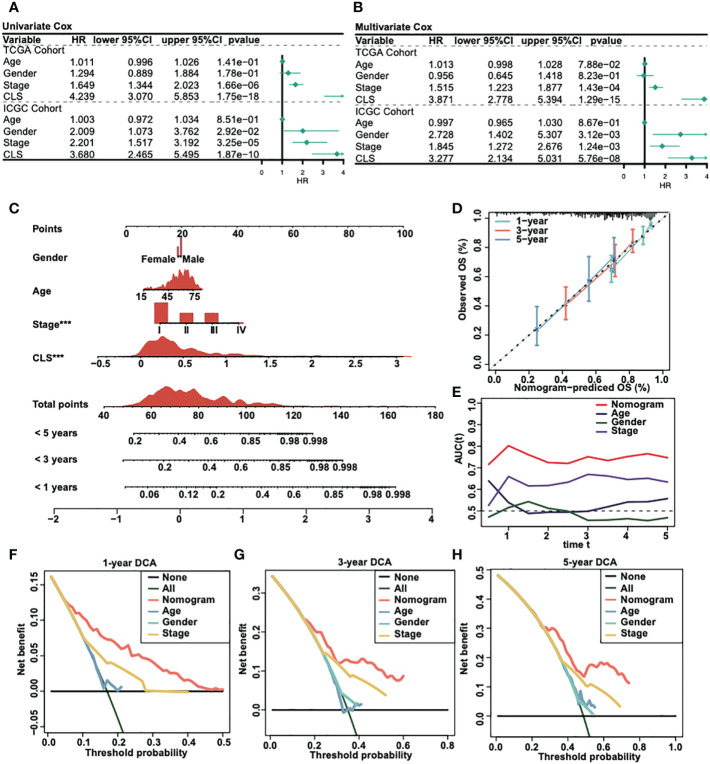
Construction of a CLS-based nomogram. **(A)** Univariate Cox regression in TCGA and ICGC cohorts. **(B)** Multivariate Cox regression in TCGA and ICGC cohorts. **(C)** Construction of a nomogram by various parameters. **(D)** Calibration curve of the CLS-based nomogram. **(E)** AUC analysis for the constructed nomogram. **(F)** One-year DCA for the nomogram. **(G)** Three-year DCA for the nomogram. **(H)** Five-year DCA for the nomogram.

We analyzed the univariate Cox regression and multivariate Cox regression in both TCGA and ICGC cohorts (**Figures 3A, B**) to figure out the possible independent prognostic factors. We announced that stage and CLS were the independent prognostic factors in HCC patients, and that the CLS was even better than stage. Thus, we created a CLS-based nomogram for HCC patients to predict the prognosis individually (**Figure 3C**). With the CLS-based nomogram, we could calculate the survival rate of less than 1-, 3- and 5-year for each HCC patient. Subsequently, we created a calibration curve to assess the accuracy of our constructed nomogram (**Figure 3D**). The calibration curves illustrated a satisfactory capacity. And the AUC of the nomogram was the largest compared to age, sex and stage (**Figure 3E**), which demonstrated that the CLS-based nomogram was stable and had a high capacity for prognostic prediction. Furthermore, we performed the 1-, 3- and 5-year DCA (**Figures 3F-H**), DCA was used to assess the usefulness of the models we interested. We evaluated the usefulness of each model by net benefit (24). In this analysis, the CLS-based nomogram showed a larger net benefit compared to other models, the result revealed that the CLS-based nomogram was worthy of application in the clinic.

### Functional analyses of the CLS model

**Figure 4:**
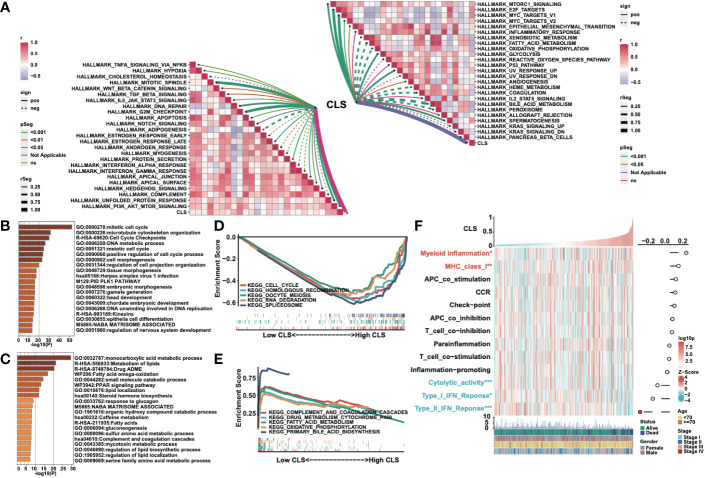
Functional analyses of the CLS model. **(A)** The correlation between CLS and the Hallmark cancer-related pathways. **(B)** The enriched items in high CLS samples in Metascape. **(C)** The enriched items in low CLS samples in Metascape. **(D)** The top five enriched items in high CLS samples by GSEA. **(E)** The top five enriched items in low CLS samples by GSEA. **(F)** The expression of the interested pathways in each sample and the correlation between interested pathways and CLS.

We built a heatmap to exhibit the correlation and the significance between CLS and hallmark gene sets (**Figure 4A**). For example, CLS had a positive correlation with MTORCI signaling with a p-value less than 0.001. In total, the majority of cancer-related pathways were significantly related to CLS, with a positive/negative correlation. Then, after obtaining the differentially expressed genes, we performed the enrichment analysis using Metascape. The top five enriched items in high CLS samples were mitotic cell cycle, microtubule cytoskeleton organization, cell cycle checkpoints, DNA metabolic process and meiotic cell cycle (**Figure 4B**). The top five enriched items in the low CLS samples were monocarboxylic acid metabolic process, metabolism of lipids, drug ADME, fatty acid omega-oxidation and small molecule catabolic process (**Figure 4C**). Furthermore, we performed GSEA to detect the pathways enriched in samples (**Figures 4D, E**). Cell cycle, homologous recombination, oocyte meiosis, RNA degradation and spliceosome were significantly enriched in high CLS samples. Complement and coagulation cascades, drug metabolism cytochrome P450, fatty acid metabolism, oxidative phosphorylation and primary bile acid biosynthesis were significantly enriched in low CLS samples. Moreover, we built a heatmap to explore the expression and correlation of some pathways of interest (**Figure 4F**). We discovered that myeloid inflammation and MHC class I were upregulated in high CLS samples, while cytolytic activity, type I and II IFN responses were upregulated in low CLS samples. The type II IFN response, however, was negatively correlated with CLS with the most significant.

### Immune analysis of the CLS model

**Figure 5:**
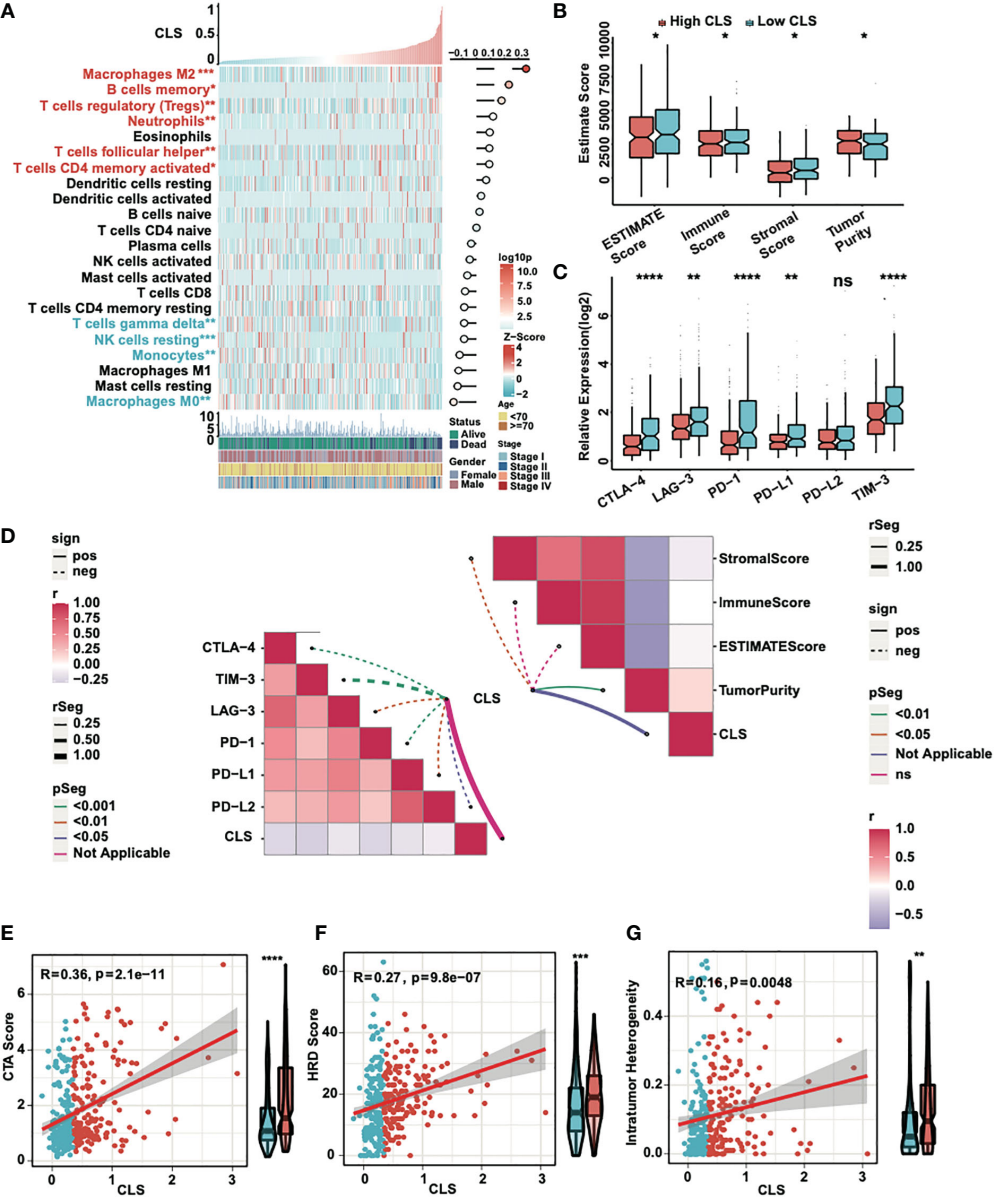
Immune analyses of the CLS model. **(A)** The expression and correlation between the TICs and CLS. **(B)** The ESTIMATE score (including immune and stromal score) and tumor purity in high and low CLS samples. **(C)** The relative expression of six immune checkpoints in high and low CLS samples. **(D)** The correlation between CLS and immune checkpoints/ESTIMATE. **(E)** The correlation between the CTA score and the CLS, and the level of CTA score in high and low CLS samples. **(F)** The correlation between the HRD score and the CLS, and the level of HRD score in high and low CLS samples. **(G)** The correlation between the intratumor heterogeneity and the CLS, and the level of the intratumor heterogeneity in high and low CLS samples. *P-value < 0.05, **P-value < 0.01, ***P-value < 0.001, ****P-value < 0.0001. ns, not significant.

First, we detected the enrichment and the correlation of the 22 TICs in samples. By generating a heatmap, we revealed that M2 macrophages, B memory cells, T regulatory cells, neutrophils, T follicular helper cells and CD4 memory activated T cells were significantly highly expressed in high CLS samples, while T gamma delta cells, NK resting cells, monocytes and M0 macrophages were upregulated in low CLS samples. Among them, M2 macrophages had the most significant positive correlation with CLS (**Figure 5A**). Then we calculated the immune and stromal scores and tumor purity (**Figure 5B**). We found that the tumor purity was higher in high CLS samples, while the immune and stromal scores were higher in low CLS samples. The results uncovered that high CLS could easily lead to tumorigenesis. In addition, we detected the relative expression of six checkpoints between high and low CLS samples (**Figure 5C**). CLTA-4, LAG-3, PD-1, PD-L1 and TIM-3 were highly expressed in low CLS samples, which indicated that low CLS patients had a better response to immunotherapy. Furthermore, a correlation between CLS and ESTIMATE/checkpoints was detected (**Figure 5D**). CLS was negatively correlated with stromal score and positively correlated with tumor purity. Nevertheless, CLS and checkpoints had a significantly negative correlation. Finally, the CTA score, HRD score and intratumor heterogeneity were evaluated. The expression of CTA was normal in the adult testis, but aberrant in several types of carcinoma (25). CTA score was associated with tumorigenesis and proliferation and was positively correlated with CLS. The CTA score was much higher in patients with high CLS (**Figure 5E**). The definition of HRD was that cells were uncapable to repair DNA double-strand breaks *via* homologous recombination repair pathway (26). As a characteristic of tumor tissue, HRD was positively correlated with CLS, and patients with high CLS had higher HRD score than patients with low CLS (**Figure 5F**). Intratumor heterogeneity, one of the reasons for the failure of cancer treatment and the determinative factor of the tumor microenvironment (27), was positively correlated with CLS. Intratumor heterogeneity was higher in high CLS patients (**Figure 5G**). Above all, patients with high CLS may have higher invasive and treatment resistance.

### Mutational analysis of the CLS model

**Figure 6:**
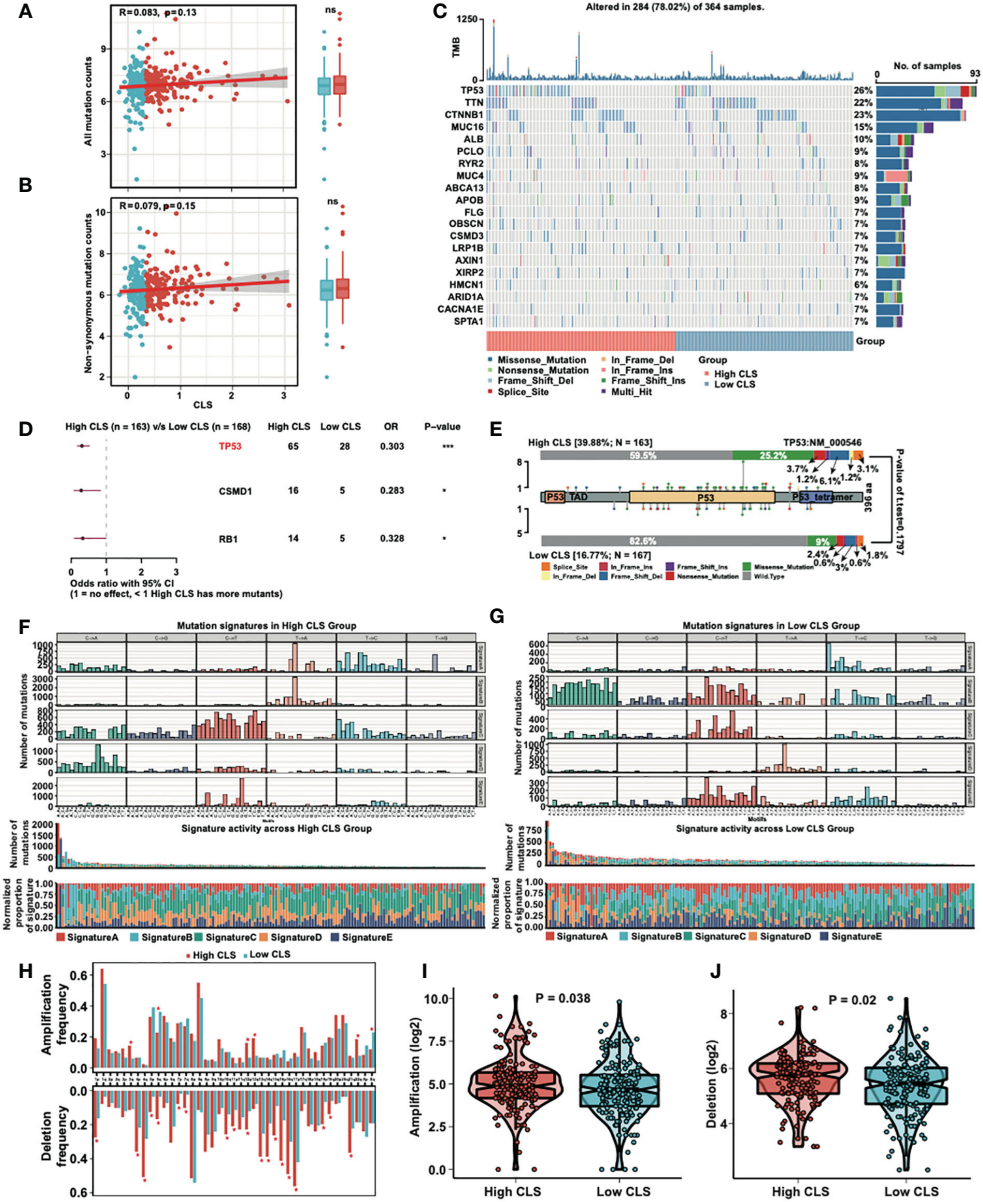
Mutational analyses of the CLS model. **(A)** The correlation between the all mutation counts and the CLS, and the number of all mutation counts in high and low CLS samples. **(B)** The correlation between the non-synonymous mutation counts and the CLS, and the number of all mutation counts in high and low CLS samples. **(C)** The waterfall plot of the top 20 altered mutation in high and low CLS samples. **(D)** The differentially mutated genes between high and low CLS samples. **(E)** The proportion and the types of the TP53 mutation in high and low CLS samples. **(F)** The number of mutations in five mutational signatures in high CLS samples. **(G)** The number of mutations in five mutational signatures in low CLS samples. **(H)** The amplification and deletion frequency in each arms between high and low CLS samples. **(I)** The total frequency of amplification in high and low CLS samples. **(J)** The total frequency of deletion in high and low CLS samples. TMB, Tumor mutational burden.

We detected the correlation and mutation counts in high and low CLS samples. However, we did not find any significance in all mutation counts (**Figure 6A**) and non-synonymous mutation counts (**Figure 6B**). Then, we exhibited a mutational waterfall plot in high and low CLS samples, and the top 20 genes with the most mutational frequency are listed (**Figure 6C**). The most frequently mutated gene was *TP53* in all samples (26%), followed by *TTN* (22%) and *CTNNB1* (23%). In addition, we compared the mutants between high and low CLS samples (**Figure 6D**). The results revealed that *TP53*, *CSMD1* and *RB1* had more mutants in high CLS samples. Since *TP53* was found to be the most significantly mutated gene between the two groups, we illustrated the mutational types of *TP53* in high and low CLS samples by generating a lollipop chart (**Figure 6E**). We found that 25.2% of mutations in high CLS samples were missense mutations, which was only 9% in low CLS samples. The percentage of other mutational types was higher in high CLS samples. Subsequently, we analyzed the mutation signatures in the two groups. By comparing five mutational signatures, we found that a difference existed between high and low CLS samples (**Figures 6F, G**). For instance, in signature B, many mutations occurred in the low CLS group but not in the high CLS group. In addition, we detected the frequency of amplification and deletion in each arm (**Figure 6H**). The results indicated that many deletions were existed in high CLS samples. In arms 3q, 12p, 12q and 22q, the mutational frequency of amplification was higher in high CLS samples but lower in the 5q and Xq arms. By detecting the total frequency of amplification (**Figure 6I**) and deletion (**Figure 6J**), we revealed that samples with high CLS showed higher amplification and deletion frequencies.

### Application of the CLS model in clinical treatment

**Figure 7:**
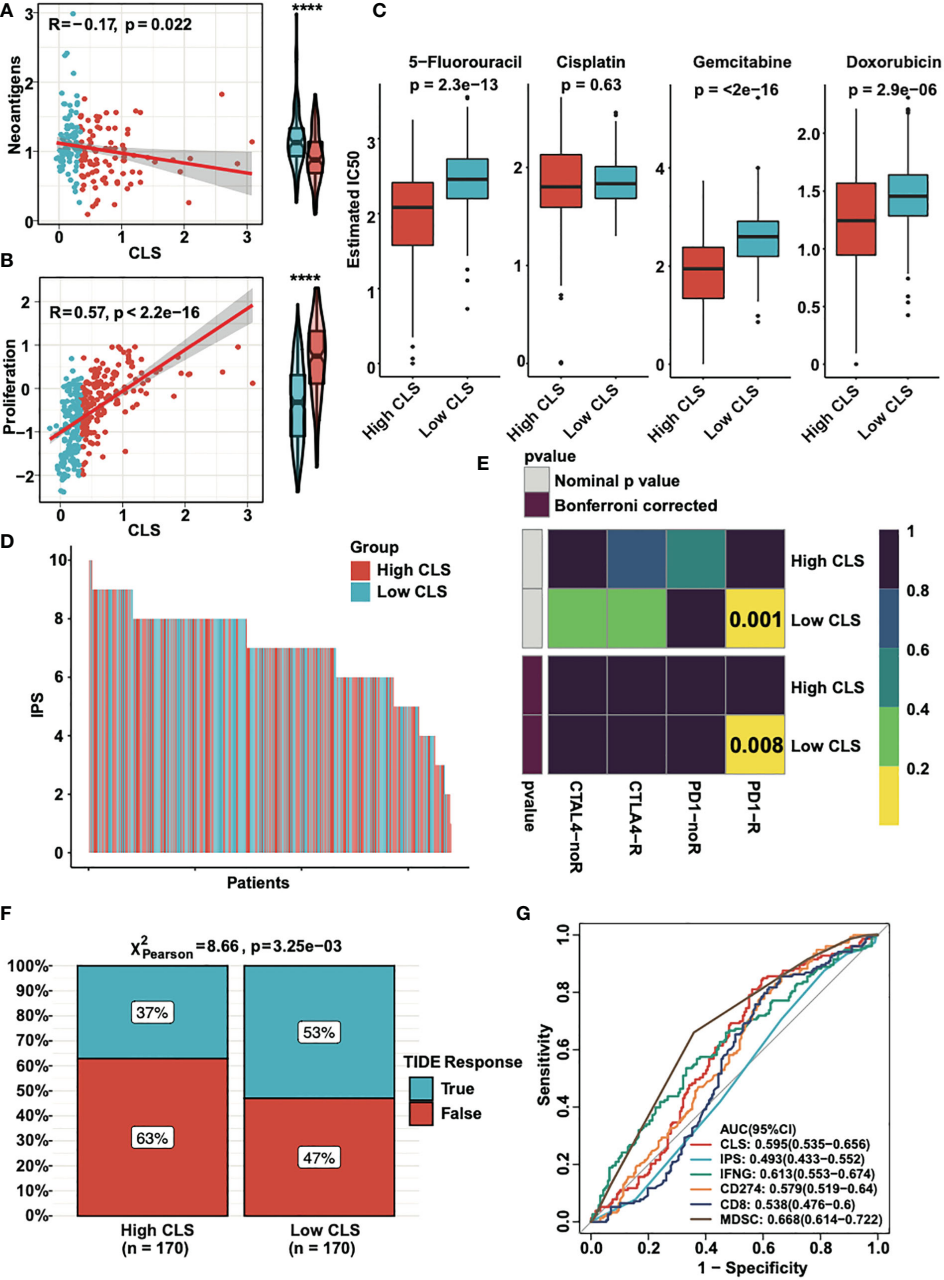
The clinical application of CLS model. **(A)** The expression and the correlation of the neoantigens in high and low CLS samples. **(B)** The expression and correlation of the proliferation score in high and low CLS samples. **(C)** The estimated IC50 of 5-fluorouracil, cisplatin, gemcitabine and doxorubicin in high and low CLS samples. **(D)** The IPS of each patients with high or low CLS. **(E)** TIDE analysis of the PD1 and CTLA4 response in patients with high and low CLS. **(F)** The proportion of the TIDE response in high and low CLS patients. **(G)** The AUC analysis of the CLS and biomarkers. IPS, Immunophenoscore.

Neoantigens, which are specifically expressed in tumor tissue, have been proved to be the vital T cell-mediated immunotherapy targets for tumor patients (28). The expression of neoantigens were detected in high and low CLS samples (**Figure 7A**). We observed a negative correlation between CLS and neoantigens; moreover, the neoantigens was upregulated in low CLS samples. The results demonstrated that the patients with low CLS may have a satisfactory response to immunotherapy. By detecting the proliferation score, we concluded that the correlation was significantly positive between CLS and proliferation, and the proliferation score was higher in high CLS samples (**Figure 7B**), which indicated that high CLS patients had a higher capacity of proliferation. Next, we detected the estimated IC50 of four chemotherapeutic drugs, which are normally used in HCC treatment (**Figure 7C**). The results showed that patients with high CLS had better sensitivity to 5-fluorourcil, gemcitabine and doxorubicin in the TCGA dataset. The same result was obtained in the ICGC dataset (**Figure S2A**). In addition, we calculated the IPS in each patient in two datasets (**Figures 7D**, **S2B**). The results showed that the low CLS patients had a higher IPS, which indicated that patients with low CLS may have a better response against immunotherapy. Moreover, the subclass mapping displayed that patients with low CLS had a better PD-1 response (**Figure 7E**), and a similar result was found in the ICGC dataset (**Figure S2C**). Furthermore, we used the TIDE algorithm to predict the immunotherapeutic sensitivity, and we detected the response rate in two subgroups in the TCGA dataset (**Figure 7F**). Patients with a low CLS had a better percentage of response than those with a high CLS. In the ICGC cohort, however, the response rate was higher in low CLS patients, with a P-value = 0.05 (**Figure S2D**). Finally, we performed the ROC analysis to compare our CLS model to five widely utilized biomarkers in the TCGA (**Figure 7G**) and ICGC databases (**Figure S2E**). The results uncovered that the CLS model had great accuracy for immunotherapeutic prediction and may act as a novel biomarker for HCC patients. Moreover, we predicted some potential small molecule drugs with related mechanisms by using MoA analysis (**Figure S3**), and the results may lead us to identify possible therapeutic methods for HCC patients.

## Discussion

In our study, we constructed and validated a novel prognostic signature based on CLS for HCC patients. We assessed our CLS model systematically. In the functional assessment, we confirmed that CLS had a high correlation with cancer-related pathways. In addition, cell cycle and immune related pathways were enriched. By performing immune analysis, we announced that the tumor characteristics were more obvious in high CLS samples, which was related to invasion and resistance to the treatment. In mutational evaluation, more mutational frequency was found in high CLS samples, and the same went for amplification and deletion. We utilized our CLS model for predicting the clinical treatment response. We revealed that 5-florouracil, gemcitabine and doxorubicin had more sensitivity in high CLS patients. Nevertheless, patients with low CLS showed a better response to immunotherapy.

Thirteen lncRNAs were selected and was verified to be highly expressed in hepatocellular carcinoma. A previous article also reported that C10orf91 was upregulated in HCC and correlated with poor prognosis (29). One published article demonstrated that the lncRNA CECR7 was upregulated in HCC and related to OS (30). Other published research uncovered that lncRNA SNHG4 was highly expressed in liver cancer tissues compared to normal liver tissues; moreover, the expression of lncRNA SNHG4 was associated with OS (31). LncRNA BPESC1 was also reported to correlate with OS, and HCC patients with high expression of BPESC1 had worse OS (32).

By performing correlation analysis, we revealed that CLS was highly correlated with some cancer-related pathways, such as mitotic spindle, DNA repair, G2/M checkpoint, PI3K-AKT-MTOR signaling, MTORC1 signaling, E2F targets and MYC targets. The source of our CLS model was the cuproptosis-related lncRNAs, which had a high correlation with the level of copper. Currently, studies have proven that the level of copper correlates with various biochemical processes. One published article pointed out that a high level of copper enhanced the drug resistance and was involved in DNA damage repair in cancer cells (33). One previous article demonstrated that copper accumulation reduced the proportion of cells in G2/M phase *via* Ras/PI3K/Akt signaling (34). In addition, another article reported that a novel copper nanocomplex inhibited cell proliferation and caused the cell death *via* the PI3K/AKT/mTOR signaling pathway in cervical cancer cells (35). These results were consistent with our findings.

We analyzed the tumor microenvironment and the enrichment of TICs in each sample. Many tumor immune cells were enriched. M2 macrophages, for example, were reported to have tumor-promoting activities promoting cell proliferation, migration, angiogenesis and immunosuppression, subsequently resulting in poor outcome of HCC (36). This result coincided with our findings that M2 macrophages were significantly enriched in high CLS samples, which had unfavorable outcomes of HCC. Previous research illustrated that infiltration of regulatory T cells inhibit the anti-tumor immune response and is correlated to unsatisfactory prognosis (37). Neutrophils have been proved to promote the progress of tumorigenesis and associated to poor prognosis (38). Our result showed that regulatory T cells and neutrophils were enriched in high CLS patients, which was a good explanation of high CLS patients with a poor overall survival. According to the analysis of the tumor microenvironment, the tumor purity was higher and the immune and stromal scores were lower in the high CLS samples. The result was corresponded to the findings that the high CLS patients had higher progression of HCC and worse survival status. By detecting the relative expression, we revealed that immune checkpoints were highly expressed in low CLS samples except PD-L2. The results indicated that the checkpoint inhibitors may have a better response in low CLS patients. In addition, in the analysis of clinical application of this article, we predicted the effect of chemotherapy and immunotherapy in high and low CLS patients. The results demonstrated that the chemotherapy was sensitive in high CLS patients, while immunotherapy was better in low CLS patients. The reversed result can be explained by the treatment chosen according to the progress of the HCC. Low CLS patients may be in the early stage of the HCC, patients may get more benefits from immunotherapy because of the easier mobilization of the immune system. However, the effect of immunotherapy may decreased in advanced HCC patients because of the immune escape and T cell exhaustion. Moreover, in TIDE analysis, the response of PD-1 and CTLA4 was better in low CLS patients with HCC. Currently, some immunotherapy trials have been performed, which have shown similar results. One of them demonstrated that anti-CTLA-4 monoclonal antibody had promising outcomes in HCC patients (39). Another study reported that an antibody against PD-1 was well tolerated and had an acceptable objective response rate (40). In addition, the combination of an anti-CTLA-4 monoclonal antibody (tremelimumab) and an anti-PD-L1 monoclonal antibody (durvalumab) was found to be tolerable and enhanced the antitumor effect (41). Overall, the immunotherapy is a potential method for HCC patients, especially for the patients with low CLS.

We know that instability of the gene is one of the characteristic of most carcinomas. Mutation drives the occurrence and development of the most type of cancers (42). In our study, we revealed three genes that had more mutations in high CLS samples. *TP53*, which is the one of the most frequently mutated genes in HCC, plays a vital role in apoptosis and cell cycle regulation (43). Studies have indicated that *TP53* mutation may cause cancer progression (44). Moreover, patients with mutated *TP53* had worse OS and relapse-free survival times (45). *CSMD1* is considered to be a tumor suppressor gene in many types of cancer, such as breast cancer (46), colorectal cancer (47), gastric cancer (48) and HCC (49); thus, the mutation of the *CSMD1* may cause the proliferation of the cancer. One published article revealed that the mutation of *CSMD1* may promote the progression of esophageal cancer (50). Interestingly, one article demonstrated that *CSMD1* mutation co-occurred with *TP53* mutation (51). In our research, we also detected the concurrent mutation of *TP53* and *CSMD1* in high CLS samples. As a tumor suppressor gene, *RB1* is a negative regulator in the progression of the cell cycle *via* the regulation of the E2F transcription factors (52, 53). Mutation of *RB1* may cause cancer genesis (54). Together, the result was sensible that the patients with a high frequency of mutated genes *TP53*, *CSMD1* and *RB1* may have a worse survival status.

We were aware of the study having some limitations. First, our results were obtained based on the online databases, and clinical trials with large samples are necessary. Second, we could not find the immunotherapy information for HCC; instead, we verified the results in a melanoma cohort. Thus, a novel HCC cohort is needed for the further analyses.

In this article, we established and verified a novel prognostic CLS model by machine learning. Meanwhile, We performed systematic analyses, including function, mutation, immunity and clinical application, to ensure the stability and value of the constructed model for the purpose of utilization of our model in the clinical assessment and treatment.

## Data availability statement

The original contributions presented in the study are included in the article/**Supplementary Material**. Further inquiries can be directed to the corresponding author.

## Author contributions

DL, JL: designed the study, analyzed the data and wrote the manuscript. HC, QM, FW, FX: assisted with the data analyses. YH: Reviewed and revised the manuscript. All authors contributed to the article and approved the submitted version.

## Supplementary material

Supplementary Figure 1 Prognostic signature based on CLS in ICGC database. A. The AUC of CLS, age, gender and stage in ICGC database. B. The survival status and the expression of the 13 cuproptosis-related lncRNAs of each sample ranked from high to low CLS in ICGC database. C. Kaplan-Meier analysis of the high and low CLS patients in ICGC database. D. The 1-, 3- and 5-year AUC of the CLS-based prognostic signature in ICGC.

Supplementary Figure 2 The clinical application of CLS model in ICGC database. A. The estimated IC50 of 5-fluorouracil, cisplatin, gemcitabine and doxorubicin in high and low CLS samples in ICGC. B. The IPS of each patients with high or low CLS in ICGC. C. TIDE analysis of the PD1 and CTLA4 response in patients with high and low CLS in ICGC. D. The proportion of the TIDE response in high and low CLS patients in ICGC. E. The AUC analysis of the CLS and biomarkers in ICGC.

Supplementary Figure 3 MoA analysis in HCC.
