# Supplementary figures and images for: Construction and systematic evaluation of a machine learning-based cuproptosis-related lncRNA score signature to predict the response to immunotherapy in hepatocellular carcinoma
Source: Front Immunol. 2023 Jan 25;14:1097075. doi: 10.3389/fimmu.2023.1097075 (PMC9905126; doi:10.3389/fimmu.2023.1097075)

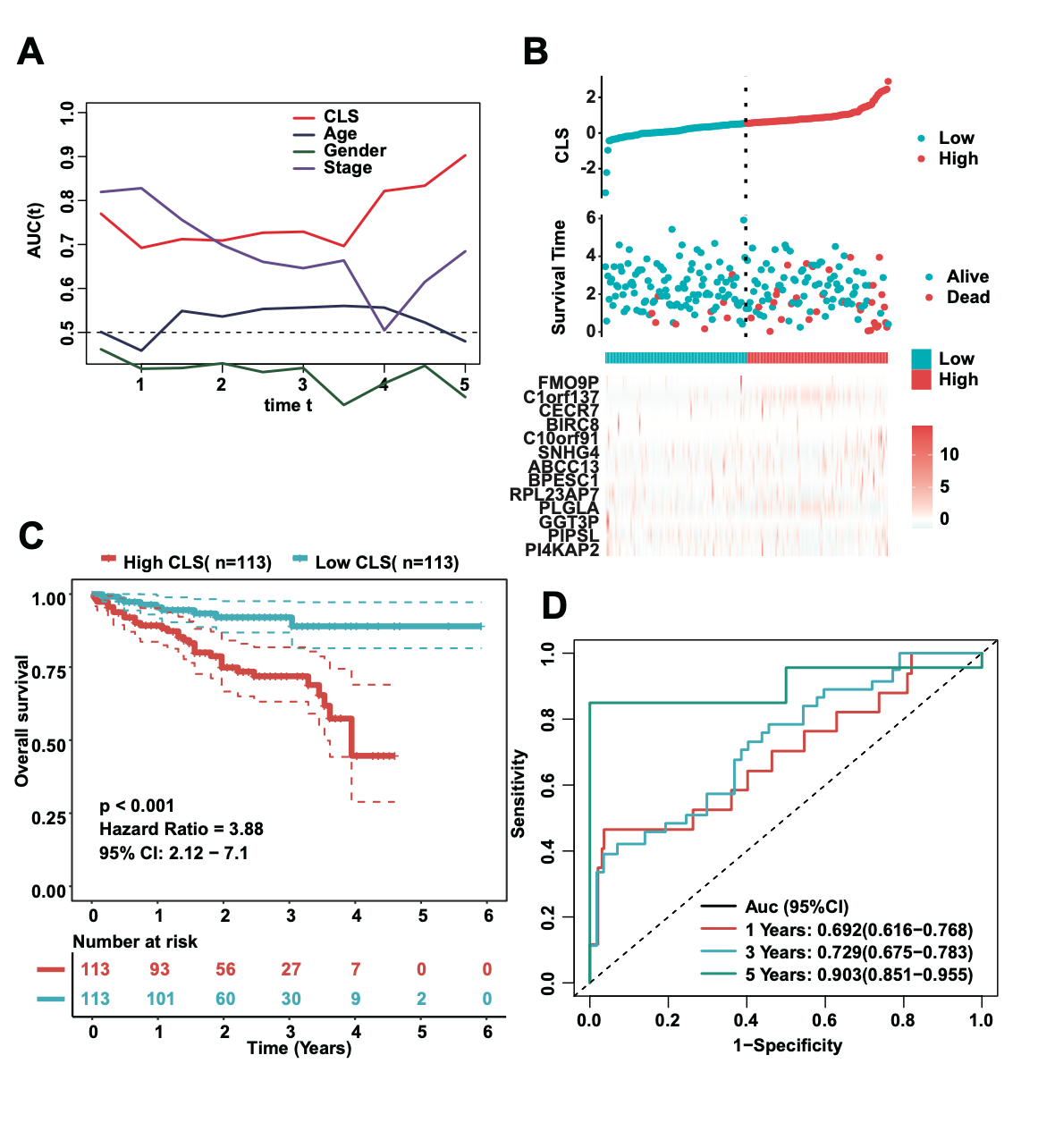

Supplement: Supplementary Figure 1 — Prognostic signature based on CLS in ICGC database. A. The AUC of CLS, age, gender and stage in ICGC database. B. The survival status and the expression of the 13 cuproptosis-related lncRNAs of each sample ranked from high to low CLS in ICGC database. C. Kaplan-Meier analysis of the high and low CLS patients in ICGC database. D. The 1-, 3- and 5-year AUC of the CLS-based prognostic signature in ICGC. [file Image_1.tiff]

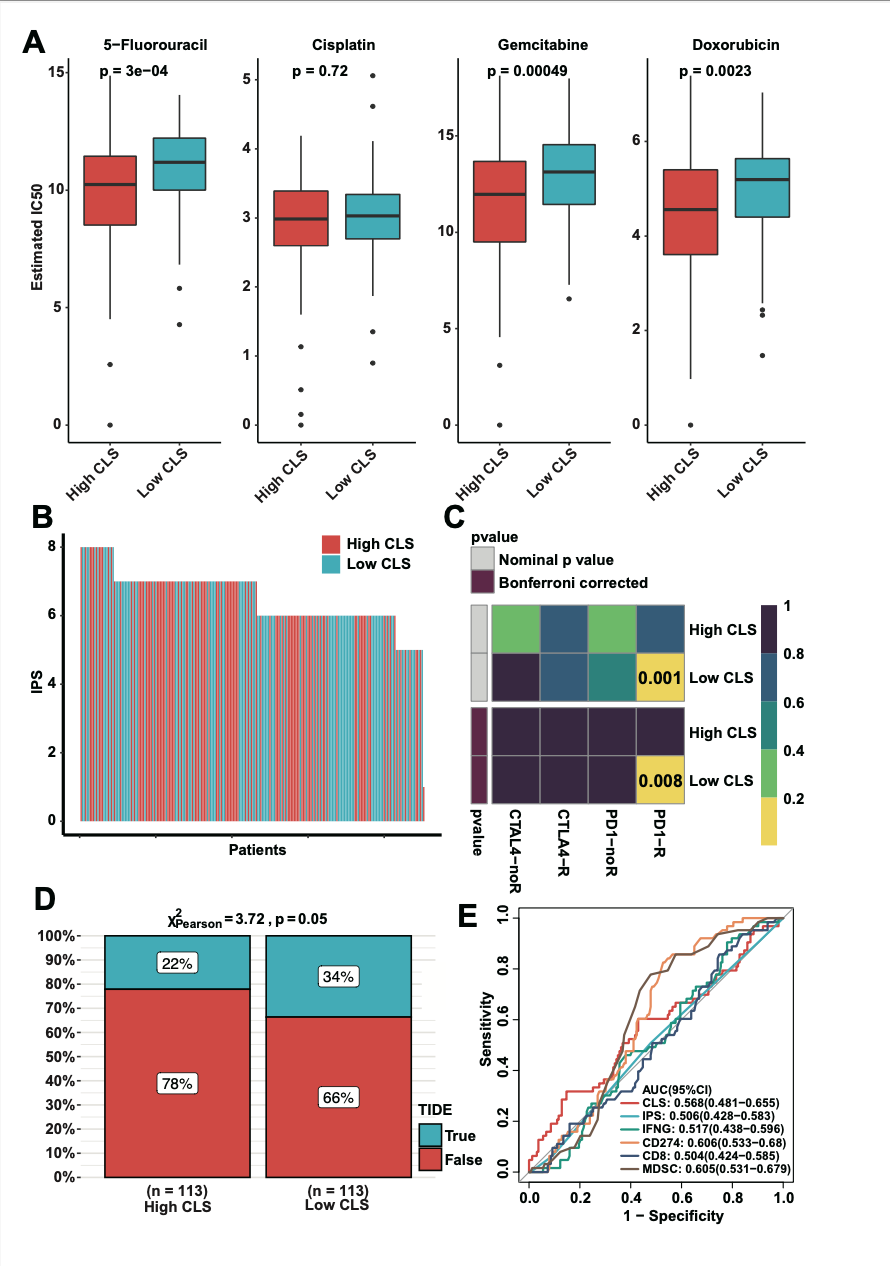

Supplement: Supplementary Figure 2 — The clinical application of CLS model in ICGC database. A. The estimated IC50 of 5-fluorouracil, cisplatin, gemcitabine and doxorubicin in high and low CLS samples in ICGC. B. The IPS of each patients with high or low CLS in ICGC. C. TIDE analysis of the PD1 and CTLA4 response in patients with high and low CLS in ICGC. D. The proportion of the TIDE response in high and low CLS patients in ICGC. E. The AUC analysis of the CLS and biomarkers in ICGC. [file Image_2.tiff]

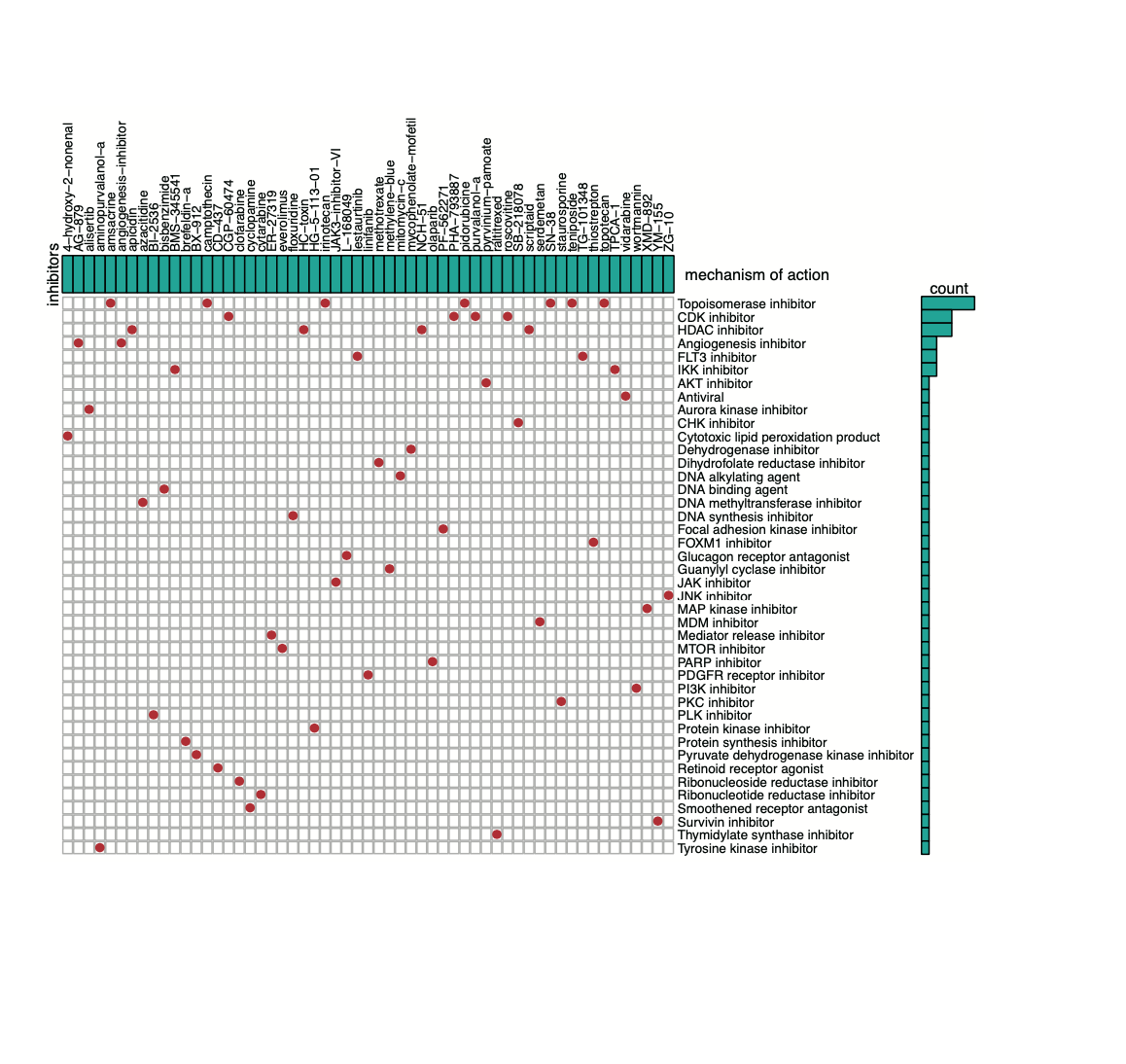

Supplement: Supplementary Figure 3 — MoA analysis in HCC. [file Image_3.tiff]
