# Supplementary material for: Construction and systematic evaluation of a machine learning-based cuproptosis-related lncRNA score signature to predict the response to immunotherapy in hepatocellular carcinoma
Source: Front Immunol. 2023 Jan 25;14:1097075. doi: 10.3389/fimmu.2023.1097075 (PMC9905126; doi:10.3389/fimmu.2023.1097075)
Supplement: Supplementary file 4 [file Table_1.docx]

**Table 1. The list of the cuproptosis-associated genes.**

| **Gene** | **Full name** |
| --- | --- |
| DLAT | Dihydrolipoamide S-acetyltransferase |
| DLD | Dihydrolipoamide Dehydrogenase |
| GLS | Glutaminase |
| LIPT1 | Lipoyltransferase 1 |
| MTF1 | Metal Regulatory Transcription Factor 1 |
| PDHB | Pyruvate Dehydrogenase E1 Subunit Beta |
| CDKN2A | Cyclin Dependent Kinase Inhibitor 2A |
| FDX1 | Ferredoxin 1 |
| PDHA1 | Pyruvate Dehydrogenase E1 Subunit Alpha 1 |
| LIAS | Lipoic Acid Synthetase |
