# Supplementary material for: Construction and systematic evaluation of a machine learning-based cuproptosis-related lncRNA score signature to predict the response to immunotherapy in hepatocellular carcinoma
Source: Front Immunol. 2023 Jan 25;14:1097075. doi: 10.3389/fimmu.2023.1097075 (PMC9905126; doi:10.3389/fimmu.2023.1097075)
Supplement: Supplementary file 5 [file Table_2.docx]

**Table S2. The sequences of the lncRNA primers**

| **Primer** | **Forward** | **Reverse** |
| --- | --- | --- |
| FMO9P | TCGGGATGTATGCCACACACTTTG | AATCTGGGTGCTTCCTCACACTTTG |
| C1orf137 | AGAACCCAGGAATGGCGTTAAAGAC | GGAAGGAGGACTGATGGTGATGTTG |
| CECR7 | ACCGTCTGTCTCTTCCTTCACTACC | ACGTGCTTATGAACAACCTGTCTGG |
| BIRC8 | TTCCTCTTTTCTTGCTGACCCTTCG | AATGCCAAACCCTGCCCTAAGC |
| C10orf91 | ACCACAGGCACCCAGGATAACTC | GGCGACGTGCTTCTTGTCTGAG |
| SNHG4 | AACTCCTGACCTTGCGATTTGCC | GAGGTTGTAGTGAGCCGAGATTGC |
| ABCC13 | CTCCACACCTTGCCTCCTCCAG | AGTCCTTGCCTTCCAGTCCTAGC |
| BPESC1 | TCCACCCTTCCTGCCTCATGTC | CCCATCATCTTTGCTGTCCCATCTC |
| RPL23AP7 | AGGGAGAGAAGTGGATTTGGGAGAC | CTTTGGCTTCGGCTTTAGGAGGAG |
| PLGLA | CCACTTCTCCCCGCAGACCTAG | GAACCAATCCCTCACAGACACAGAG |
| GGT3P | TGGAGATTGGGAGGGACACACTG | GGTGCTGTTGTAGATGGTGAGGAAG |
| PIPSL | CACCTGTTGCCTTCCGCTACTTC | CTCATCGTCGCTGGACACATAGAAC |
| PI4KAP2 | AAGGTGAAGCGATGTGGAGTTAGTG | GGCAGTCGTCTCCCAGTTTGAAG |
